# The Role of Helminthophagous Fungi in the Biological Control of Human and Zoonotic Intestinal Helminths

**DOI:** 10.3390/pathogens13090741

**Published:** 2024-08-30

**Authors:** Jackson Victor de Araújo, Júlia dos Santos Fonseca, Beatriz Bacelar Barbosa, Helbert Ananias Valverde, Huarrisson Azevedo Santos, Fabio Ribeiro Braga

**Affiliations:** 1 Department of Veterinary Medicine, Federal University of Viçosa—UFV, Viçosa 36570-900, MG, Brazil; jvictor@ufv.br (J.V.d.A.); helbert.valverde@ufv.br (H.A.V.); 2 Department of Epidemiology and Public Health, Federal Rural University of Rio de Janeiro—UFRRJ, Seropédica 23890-000, RJ, Brazil; beatrizbacelar_@hotmail.com (B.B.B.); huarrisson@yahoo.com.br (H.A.S.); 3 Laboratory of Experimental Parasitology and Biological Control, Vila Velha University—UVV, Vila Velha 29102-920, ES, Brazil; fabio.braga@uvv.br

**Keywords:** helminthosis, helminthophagous fungi, humans, One Health, parasitic nematodes, public health

## Abstract

Nematophagous, or helminthophagous fungi of the genera *Duddingtonia*, *Arthrobotrys*, *Monacrosporium*, *Pochonia*, *Paecilomyces*, and *Mucor*, have been used over the years in in vitro and in vivo experiments to control helminth parasites that are potentially zoonotic. These fungi have shown efficacy against the following helminth genera: *Ancylostoma*, *Toxocara*, *Enterobius*, *Strongyloides*, *Angiostrongylus*, *Taenia*, *Fasciola*, and *Schistosoma*. The results obtained from these experiments, together with studies on soil contamination, suggest the viability of their use as a sustainable and effective strategy to reduce environmental contamination by these zoonotic parasites. Therefore, the aim of this review was to address the role of helminthophagous fungi in the biological control of potentially zoonotic helminths. To this end, we describe (1) a brief history of helminthophagous fungi; (2) a discussion of some potentially zoonotic intestinal parasites; (3) the importance of helminthophagous fungi in the control of nematodes, cestodes, and trematodes; and (4) the potential of helminthophagous fungi as a practical and sustainable strategy.

## 1. Introduction

In various parts of the world, zoonotic diseases caused by helminths are currently a constant threat to public health [1]. Helminth infections are among the most common infections worldwide, with an estimated 1.5 billion infected people, or 24% of the world’s population [2]. These infections generate many impacts on human and animal health, ranging from gastrointestinal disorders to more serious systemic diseases. Helminth parasites, which include various types of worms, such as trematode (*Clonorchis* spp., *Echinostoma* spp., *Metagonimus* spp., *Nanophyetus* spp., *Heterophyes* spp., *Fasciola* spp., *Schistosoma* spp., *Paragonimus* spp., and *Opisthorchis* spp.), cestode (*Dipylidium* spp., *Diphyllobothrum* spp. *Echinococcus* spp., *Hymenolepsis* spp., and *Taenia* spp.), and nematode (*Ascaris* spp., *Ancylostoma* spp., *Anisakis* spp., *Capillaria* spp., *Gnathostoma* spp., *Necator* spp., *Parastrongylus* spp. *Strongyloides* spp., and *Trichinella* spp.), have been associated with a wide range of human diseases. Ecological, environmental, and socioeconomic factors influence the successful transmission of these parasites between animals and humans [1,3].

The use of antihelminthic drugs still represents the most significant approach to controlling helminth infections in animals and humans [4]. The use of these drugs has significantly reduced the prevalence of these infections in many parts of the world [5]. However, misuse and often unplanned use have raised concerns about the emergence of resistance and environmental contamination. In terms of the environment, one of the main concerns is the effect of antihelminthics on nontarget organisms, causing an ecological imbalance that significantly hampers efforts to effectively control helminthic infections [6].

Given the challenges associated with controlling zoonotic helminths, several studies are investigating alternative strategies to control these parasites, including biological control using helminthophagous fungi. The mechanisms by which these fungi trap, infect, and prey helminths provide an additional tool for sustainable and eco-friendly helminth control. The rationale for using helminthophagous fungi in biological control is that they act on the free life stages of helminths present in the environment, interrupting their life cycle and reducing the risk of transmission to humans and animals [7,8,9,10].

One Health promotes an integrated vision of health, recognizing that human, animal, and environmental health are inextricably linked. Controlling diseases caused by zoonotic helminths through a One Health approach necessarily promotes collaboration between veterinarians, physicians, biologists, and other specialists with a holistic approach to prevention and treatment [11]. A key component of this approach is biological control. Rather than relying solely on chemical treatments, biological control uses living organisms to reduce helminth populations. For example, introducing helminthophagous fungi or using beneficial microorganisms that compete with the parasites can be an effective strategy. This not only reduces the parasite burden but also minimizes negative impacts on the environment and the health of other living organisms [12,13]. Biological control using helminthophagous fungi can be applied based on knowledge of the ecology and epidemiology of helminths, which in turn are influenced by environmental conditions and livestock management practices. By recognizing and addressing these linkages, we can develop and implement more effective and sustainable solutions to reduce the impact of helminth infections, thereby protecting human, animal, and environmental health in a holistic manner [14,15].

Experiments have demonstrated the efficacy of various fungi, including *Pochonia chlamydosporia*, *Duddingtonia flagrans*, *Arthrobotrys* sp., *Paecylomices* sp., *Monacrosporium* sp., *Fusarium solani,* and *Mucor circinelloides*, in trapping and reducing infective forms of helminths [10,16,17,18,19,20,21,22,23]. These findings, along with soil contamination studies, suggest the feasibility of utilizing helminthophagous fungi as a sustainable and effective environmental control strategy. Despite the growing interest in helminthophagous fungi as potential biocontrol agents, several knowledge gaps and research challenges remain. These include elucidating the mechanisms underlying fungal predation and ovicidal activity, optimizing the efficacy of fungal formulations under diverse environmental conditions, and assessing the long-term ecological consequences of fungal interventions. Addressing these gaps will be essential for advancing our understanding of the role of helminthophagous fungi in helminth control and translating this knowledge into effective public health interventions.

In this context, helminthophagous fungi have emerged as a potential tool for the control of environmental forms of helminths. The purpose of this review is to emphasize the importance of these fungi in the control of free-living forms of helminth parasites in humans by highlighting the research that has been conducted for this purpose.

## 2. Helminthophagous Fungi—A Historical Report

The first research on the antagonism of helminthophagous fungi against animal parasitic nematodes was conducted in France by Deschiens (1939a,b), Descazeaux and Capelle (1939), Deschiens (1939c), Deschiens (1941), Roubaud and Descazeaux (1939), Roubaud and Deschiens (1939), and Roubaud and Deschiens (1941) [24,25,26,27,28,29,30,31]. During the 1970s, scientists focused on studying biological control methods to manage nematodes that posed a threat to agriculture. One particular area of research involved investigating the use of fungi, sometimes referred to as nematode “eaters”, or helminthophagous, which attracted significant attention [32]. In the 1950s, in Russia, Soprunov and Tendetnik (1957) [33] controlled yellows caused by *Ancylostoma duodenale* in gold miners by spraying *Arthrobotrys* spp. at a dosage of 100–150 conidia/m^2^. Lysek (1978) [34] made another report on the activity of helminthophagous fungi against human parasitic helminth targets after a hiatus of almost two decades. Lysek (1978) and Lysek and Sterba (1991) [19,34] talked about how the helminthophagous fungus *Pochonia chlamydosporia* (syn = *Verticilium chlamydosporium*) killed *Ascaris lumbricoides* eggs in a type 3 way, which meant that the eggs were destroyed. Frassy et al. (2010) [8] define the type 3 effect as the lytic effect, which includes changes in the structure of the embryo and shell, as well as the penetration of hyphae and colonization of the interior contents of the egg. During this instance, they explained that the eggs’ “infection” could originate from their shell, which contains a significant amount of chitin and proteins. Since the 1990s, these authors’ findings have provided insight into the possible impact of fungi on many types of human helminth parasites, including those that can be transmitted between animals and humans. Due to their significant biological activity, including predation, infection, and ovicidal activity, helmintophagous fungi remain a viable long-term management alternative in contaminated environments [32].

## 3. Intestinal Parasites Transmitted between Animals and Humans

### 3.1. Dogs and Cats

**Figure 1:**
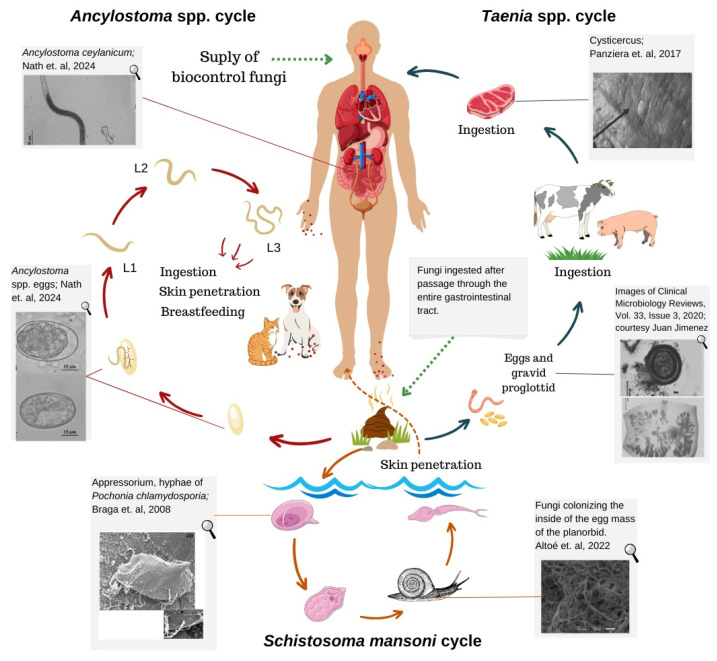
Dynamics of the use of helminthophagous fungi for the biocontrol of helminths in humans and zoonotic intestinal parasites (Nath et al. 2024; Braga et al. 2008; Altoé et al. 2022; Panziera et al. 2017) [16,17,18,37,38,39]. The illustrations present in the *S. mansoni* cycle came from Servier medical Art, being licensed under CC BY 4.0: https://smart.servier.com.

The proximity between humans and pets is increasing. Since the COVID-19 pandemic, this contact has become even more intense, with an increase in the number and time people spend at home, as well as an increase in the number of adoptions [35]. This interaction, often combined with community misinformation about zoonotic diseases transmitted between animals and humans, can lead to pathogen transmission (Figure 1) [36].

Fonseca et al. (2024) [40] and Ayinmode et al. (2018) [41] have found that dogs have a significant role in the spread of parasites that can harm humans. Hence, it is crucial to implement methods to regulate these diseases in these animals to safeguard the health of the community. *Ancylostoma caninum* and *Toxocara canis* are the most significant helminths in the connection between dogs and people. The routes of infection for *Ancylostoma caninum* include the ingestion of infective larvae and the penetration of the parasite through the skin. In contrast, infection by *Toxocara canis* can occur through various pathways: ingestion of larvated eggs containing the infective form, transplacental transmission, transmammary transmission, or ingestion of paratenic hosts, such as rodents, birds, and other animals. These are the causative agents of zoonotic diseases, specifically referred to as visceral larva migrans and cutaneous larva migrans. Furthermore, *Dipylidium caninum*, *Echinococcus* spp., *Strongyloides stercoralis*, and *Spirometra* ssp. are also significant parasites in this association. 

On the African continent, in a study conducted in Ibadan, Nigeria, Ayinmode et al. (2018) [41] identified the presence of *Toxocara* spp. (9.8%), *Strongyloides* spp. (3.9%), *Ancylostoma* spp., and *Uncinaria* spp. (24.6%), with a lower prevalence in street dogs, being identified in samples collected in various areas, such as playgrounds and schools. The authors in this work report that the large number of zoonotic parasite eggs in dog feces in these locations indicates that these areas are a source of infection for animals and people. Prevalences of 31.9% for *Ancylostoma*/*Uncinaria* spp., 27.4% for *Toxascaris leonina*, and 27.1% for *Toxocara canis* were found in four regions of Morocco [42] during an investigation into the presence of zoonotic intestinal parasites. In South America, another study in Talca, Chile, found a prevalence of 14% for *T. canis* in 100 dog fecal samples [43]. In a study conducted in the municipality of Taubaté, São Paulo, Brazil, feces were collected in parks frequented by dogs, revealing that 95.65% of the samples tested positive for *Ancylostoma* spp., indicating a significant risk of transmission to humans in the analyzed public area [44].

In the United States, a similar study was conducted in recreational areas where fecal samples were collected from 288 parks. The analyses showed that 7.1% of the samples contained hookworms, 1.9% trichurids, and 0.6% ascarids [45].

In northwestern Mexico, zoonotic intestinal parasites were detected in 103 stray dogs in the Mexicali Valley, an agricultural and livestock region. The most prevalent parasite was *Dipylidium caninum* (16.50%), followed by *Toxocara canis* (3.88%) and *Toxascaris leonina* (1.94%) [46].

In Uzbekistan, a survey of zoonotic helminths in 399 dogs from urban and rural areas was conducted to assess the risk to the human population. The main zoonotic parasites found were *D. caninum* (51.38%), *A. caninum* (24.06%), *Uncinaria stenocephala* (36.84%), *T. leonina* (88.72%), and *T. canis* (93.73%) [47].

Cats also have a close relationship with humans. Some helminths can be transmitted through this contact. Among these parasites, it is worth mentioning the adverse effects on human health caused by *T. canis* and *T. cati*. These parasites pose a zoonotic risk and can lead to ocular toxocariasis in people, particularly in youngsters. Additionally, they can also induce visceral larva migrans. *A. caninum*, a parasite that causes cutaneous larva migrans disease in humans, disrupts growth and nutrition by harming the lining of the intestines. This damage leads to bleeding, loss of iron, and anemia [48]. Furthermore, its prevalence remains stable over time, highlighting the necessity for improved control measures [49].

In Africa, intestinal parasites in cats were evaluated in Egypt by analyzing 143 fecal samples. The prevalences of the main parasites found were *Toxocara cati* (30.0%), *Toxascaris leonina* (22.4%), hookworms (8.4%), and *Dipylidium caninum* (0.7%) [50]. Additionally, a similar study in Ethiopia, which included 203 cats from various districts, found *T. cati* in 9.4% and *D. caninum* in 8.9% of the cats [51].

In South America, research in countries such as Brazil provides further insight. For example, a study conducted at the Instituto Municipal de Medicina Veterinária Jorge Vaitsman in Rio de Janeiro, Brazil, examined intestinal parasites in dogs and cats [52]. The study included 400 dogs and 208 cats that were treated between August 2017 and November 2018. The overall prevalence of parasites was 11.3% in dogs and 24.5% in cats. Among the zoonotic parasites, 8.3% of dogs were infected with Ancylostomatidae, making them the most common, while 12.5% of cats were infected with *D. caninum* [52]. In another study from Jataí, Goiás, Brazil, intestinal parasites were detected in 359 dogs and 55 cats. The zoonotic parasites identified were *Ancylostoma* spp., with a prevalence of 29.53% in dogs and 47.27% in cats; *Toxocara* spp. in 7.52% of dogs and 3.64% of cats; and *D. caninum* in 6.13% of dogs and 7.27% of cats [53].

In North America, the prevalence of zoonotic parasites in wild cats from Virginia, USA, was investigated, revealing the following prevalences for the main parasites: *Toxocara cati* (58.85%) and *Ancylostoma* spp. (18.75%) [54]. In another study conducted in Oklahoma, USA, the prevalence of these parasites in stray cats was as follows: *T. cati* (44.6%), *Ancylostoma* spp. (11.2%), and *Dipylidium caninum* (4.5%) [55].

In Asia, a study in Nepal investigated the prevalence of these parasites in cats and found 94.4% in domestic cats and 100% in wild cats. The study collected 90 fecal samples from domestic cats and 17 samples from wild cats. The parasites with the highest prevalence were *Ancylostoma tubaeforme* (60.70%), *Ancylostoma braziliensis* (25.20%), *T. cati* (40.20%), Taeniidae 21.50% [56]. In China, another study identified prevalences of 17.78% for *T. cati*, 6.39% for Ancylostomatidae, and 3.06% for oocysts similar to *Toxoplasma* spp. [57]. 

In Europe, in Italy, a study on intestinal parasites in 132 shelter cats from Latium and Tuscany detected prevalences of 9% for *T. cati*, 2.3% for Ancylostomatidae, and 0.7% for *Strongyloides* spp. [58]. Intestinal zoonotic parasites in cats were studied in playgrounds in southern Spain, revealing the following prevalences for the main parasites: *Ancylostoma caninum* (3.0%), *Toxocara* spp. (17.0%), *Uncinaria stenocephala* (9.0%), and *Dipylidium caninum* (3.0%) [59].

### 3.2. Swine and Cattle: Teniasis–Cysticercosis Complex

In different parts of the world, teniasis and cysticercosis are diseases of socioeconomic importance. They are defined as neglected zoonoses, especially in rural areas. *Taenia solium* and *Taenia saginata* are the etiologic agents of human teniasis. We refer to their larval forms as *Cysticercus cellulosae* in swine and *Cysticercus bovis* in cattle. The parasite’s cysticercus is transmitted to humans through the consumption of raw or undercooked pork and beef meat. The intermediate hosts for *T. solium* are pigs (animal cysticercosis) and humans (human cysticercosis), as well as cattle for *T. saginata* (Figure 1). These animals unintentionally consume the eggs from the environment, whether through soil or pasture, leading to the formation of cysticercus, which lodges in the muscles of these hosts, thereby causing cysticercosis, the parasite’s larval stage. In addition to this stage of tapeworms, there is also the free-living stage, the egg [60,61]. 

To control this complex of diseases, it is necessary to interrupt the life cycle of the parasites. There are already known ways to achieve this, such as improving basic sanitation and educating people about hygiene and caution when eating raw or undercooked meat, as well as its origin. For example, in the *Taenia solium* biological cycle, the only definitive host is the human, which is the only source of infection for pigs. Therefore, it is possible to eradicate the disease through control programs involving humans [60,61]. Accordingly, as with so many other important diseases, complementary control programs to the conventional ones already in use, such as the use of biocontrol fungi, can be implemented. Examples of this possibility are some studies such as those in [16,20,21] using the fungi *Paecilomyces lilacinus*, *Pochonia chlamydosporia*, *Duddingtonia flagrans,* and *Monacrosporium thaumasium* against the eggs of *T. saginata*.

## 4. Helminthophagous Fungi vs. Nematodes of Human Importance and Zoonotic Nematodes

Following the work of Soprunov and Tendetnik (1957) [33] and Fonseca et al. (2023), [62] demonstrated from the reports of Lysek (1978) [34] that it was possible to replicate, in the laboratory, after almost 18 years of research, the interaction and subsequent destruction of eggs of *Toxocara canis*, a geohelminth that causes visceral larva migrans in humans by the ovicidal fungus *Paecilomyces lilacinus*. This study also provided the first evidence for the production of extracellular enzymes by helminthophagous fungi, including predators, ovicides, endoparasites, and producers of toxic metabolites, knowledge that is now widely accepted.

As described above, nematodes such as *T. canis* are the subject of biological control research due to their presence in the environment and the risk they pose to human health. In this sense, Braga et al. (2007) [63] recorded the type 3 effect with the fungus *Pochonia chlamydosporia* and with isolates of predatory fungi such as *Duddingtonia flagrans* and *Monacrosporium thaumasium* on *Ascaris lumbricoides* eggs. The studies of Braga et al. (2007) [63] spurred the development of methods for environmental control of human parasitic helminths in the years that followed. Predatory fungi produce hyphae that are modified into specialized structures called traps, which capture and attach to helminths. The authors hypothesized that trap-producing fungi of the genera *Duddingtonia* and *Monacrosporium*, although having only a type 1 effect (adhesion to the eggshell) as proposed by Lysek (1978) [34], could weaken the protein and lipid components of the eggs. This hypothesis had already been proposed by Morgan-Jones (1983) [64], who mentioned the idea of giving the animals eggs previously exposed to predatory fungi to test the inviability of the eggs and the future embryo. In the following years, work on controlling human helminth infections continued in chronological order.

According to Lysek and Sterba (1991) [19], the main characteristic of an ovicidal fungus is to have a type 3 effect during the infection process of eggs and they established the following parameters: type 1, physiological effect without morphological harm to the eggshell, where hyphae are noticed adhered to the shell; type 2, lytic effect with morphological change in eggshell and embryo, without hyphae penetration through the shell; and type 3, lytic effect with morphological change in embryo and shell, as well as hyphae penetration and internal colonization of the egg.

Braga et al. (2008a,b,c) [16,17,18] reported important results for understanding ovicidal activity and possible future environmental control of zoonotic gastrointestinal parasite eggs. Braga et al. (2008b,c) [17,18] reported the first observation of predatory fungi, such as *D. flagrans*, expressing adhesion to the eggshell (type 1 effect). This discovery opened new possibilities to explore the diversity of fungal actions. In addition, Braga et al. (2009a) [65] recorded the helminthophagic activity of *P. chlamydosporia* in the eggs of *Enterobius vermicularis*, a nematode that causes anal discomfort and itching in preschool children.

Therefore, the Brazilian research groups thoroughly investigated the activity of helminthophagous fungi in the context of the One Health concept, including their predatory, ovicidal, and enzymatic actions [32]. Researchers globally have acknowledged the potential of companion animals to serve as reservoirs for zoonotic helminths [7,65].

Carvalho et al. (2009) [22] verified that *M. thaumasium* preys on infective larvae of *Ancylostoma caninum*, a nematode that affects dogs and cats and causes cutaneous larva migrans in humans. This suggests a potential future application of fungi in controlling environmental parasites.

In a significant study, Braga et al. (2010a) [66] compared the predatory capabilities of different isolates of the fungi *D. flagrans*, *M. thaumasium*, *Monacrosporium sinense*, and *Arthrobotrys robusta* on the third-stage larvae (L3) of *Ancylostoma ceylanicum*. In the same time frame, Braga et al. (2010b) [7] discovered that helminthophagous fungi were capable of capturing and destroying L3 in a controlled environment, indicating their potential as biological agents for controlling *Strongyloides stercoralis*, a parasitic worm that infects a significant fraction of the global population. Frassy et al. (2010) [8] and Carvalho et al. (2010) [9] also recorded important results on the interactions between predatory fungi and ovicides on *T. canis* under laboratory conditions, using methods that can serve as a basis for future studies.

Since 2011, a new approach to the use of helminthophagous fungi has been more intensively explored: the production of crude extracts rich in extracellular enzymes. Paula et al. (2013) [67] reported, for the first time, the destruction of *Angiostrongylus cantonensis*, an important zoonotic nematode that causes eosinophilic meningoencephalitis in humans, after interaction with predatory helminthophagous fungi. De Souza Maia Filho et al. (2013) [23] found the presence of fungi parasitizing *T. canis* eggs in the soil of southern Brazil, highlighting the ovicidal activity of the *Trichoderma* and *Fusarium solani* complexes.

In 2017, researchers published a new and third phase of research into the adaptability of helminthophagous fungi. Silva et al. (2017) [68] and Paula et al. (2013) [67] were the first to show that the fungus *D. flagrans* produces nanoparticles with nematicidal action against *A. caninum* L3. This pioneering research indicated the possibility of developing a bio-antihelminthic that might be employed in the future to manage gastrointestinal parasites that have a global impact. Viña et al. (2022) [69] found that chlamydospores from *Mucor circinelloides* and *D. flagrans* effectively reduced helminth egg production in dogs by 96–98% after treatment (Table 1). To construct Table 1, a search was carried out in the following databases: Scielo, Scopus, Pubmed, Clarivate, and Web of Science, using the following keywords: “helminthophagous fungi” versus nemathelminths.

The study by Fonseca et al. (2024) [40], which describes various in vivo and in vitro experiments demonstrating the efficacy of formulations in controlling these parasites, demonstrates that fungal formulations have shown promising results in the environmental control of zoonotic helminths in dogs. These studies are crucial for the management of helminthiases in dogs, with the consequent risk of transmission to humans. The authors also mention that there is already a commercially registered formulation based on the fungus *Duddingtonia flagrans* (BioVerm^®^—Ghenvet/Cinergis Agribusiness Ltda., Paulínia, Brazil) for the control of helminthiases in grazing animals with practical efficacy. The results of this study demonstrate the viability of future commercial formulations for the control of free-living stages of helminths in kennels, shelters, public places, etc., with promising improvements for animal health due to the reduction in the infectious load in the environment. Therefore, it serves as a sustainable alternative with benefits not only for environmental health but also for efficient control of animals. In this context, *Pochonia chlamydosporia* is a strong option for use in these formulations, as it was the most commonly used species in the in vitro experiments described. Bwalyaa et al. (2011) [72] have already recognized and demonstrated the species’ high ovicidal potential in the literature.

## 5. Helminthophagous Fungi vs. Human-Associated Platyhelminths

Zoonoses caused by helminth infections are a major public health problem [73]. These include teniasis, fascioliasis, and schistosomiasis caused by the platyhelminths *Taenia saginata*, *Taenia solium*, *Fasciola hepatica*, and *Schistosoma mansoni*. Studies investigating the predation of these worms by helminthophagous fungi have significant implications for control strategies, as shown in Table 2. To construct Table 2, a search was carried out in the following databases: Scielo, Scopus, Pubmed, Clarivate, and Web of Science, using the following keywords: “helminthophagous fungi”, “helminthophagous fungi versus flatworms”.

In light of the work included in Table 2, we can highlight the experiment by Braga et al. (2008a) [16], which demonstrated the ovicidal effect of the fungus *Paecilomyces lilacinus* on *T. saginata* eggs. The study showed 25.5% effectiveness of the fungus *P. lilacinus* on *T. saginata* eggs under laboratory conditions successfully after 10 days. Subsequently, Braga et al. (2008b,c) [17,18] conducted successful experiments on the interaction between predatory fungi, such as *Duddingtonia flagrans*, *Monacrosporium sinense*, and *Pochonia chlamydosporia*, and ovicidal agents on eggs of *Fasciola hepatica* and *S. mansoni*, helminths that cause important human diseases (fascioliasis and schistosomiasis, respectively). It was the authors of these studies who were able to see, for the first time, structures called appressoria. This brought to light the enzyme production of these helminthophagous fungi, which was still a new topic of study. Importantly, *D. flagrans* and other predatory fungi showed the ability to adhere to eggshells for the first time (type 1 effect), expanding the range of potential fungal actions.

Araujo et al. (2009) and Araujo et al. (2010) [20,21] documented the ovicidal activity of *P. chlamydosporia*, *D. flagrans*, *Monacrosporium thaumasium*, and *P. lilacinus* on *T. saginata* eggs under laboratory conditions. In the study carried out by Araujo et al. (2009) [20], the fungi *D. flagrans* and *M. thaumasium* showed a type 1 effect at 5-, 10-, and 15-day intervals, characterized by a lytic effect without significant morphological changes in the eggshell. On the other hand, the fungus *P. chlamydosporia* showed ovicidal activity, mainly through internal colonization of *T. saginata* eggs. The study by Araujo et al. (2010) [21] compared the ovicidal activities of *P. chlamydosporia* and *P. lilacinus* (type 3 effect) on *T. saginata* eggs. Both fungi showed significant ovicidal activity (*p* < 0.05) compared with the control group. However, after 15 days of incubation, *P. lilacinus* showed greater ovicidal activity (type 3 effect) compared with *P. chlamydosporia*. Overall, *P. chlamydosporia* and *P. lilacinus* demonstrated the potential for biological control of this platyhelminth. These studies have been instrumental in updating the understanding of the tapeworm–cysticercosis complex, which is a major human burden in tropical regions.

## 6. Exploring the Potential of Helminthophagous Fungus

The role of helminthophagous fungi in veterinary parasitology is expanding. Innovative, widely published research is already pointing to a promising horizon for the practical application of these organisms in human parasite control. In Brazil, for example, Bioverm^®^, the first natural product containing the fungus *Duddingtonia flagrans* (with 10^5^ chlamydospores per gram), is already available for the control of gastrointestinal parasitic nematodes in production animals [12,13]. This product has been successfully used commercially in the field, with research demonstrating its viability in different species of livestock. Thus, this commercial formulation can be considered the most modern form of integrated biological control of worms, as it leaves no residue in milk, meat, or other products of animal origin. On the other hand, an additional “key” to the practical viability of biological control in human medicine comes from experimental results demonstrating the efficacy of the association of helminthophagous fungi, such as *D. flagrans* with anthelmintics [74,75,76].

Lima et al. (2020) [76], in a groundbreaking study, looked at how different chemical compounds (including albendazole, ivermectin, glycerol, and petroleum jelly) and biological compounds (including the helminthophagous fungus *Monacrosporium thaumasium*) could be used together to control *Ancylostoma caninum*. This study obtained L3 larvae of *A. caninum* from coprocultures of feces from naturally infected dogs. The authors used 1% ivermectin, 1% albendazole, 100% glycerin, 100% Vaseline, and an isolate of *M. thaumasium* (NF34), alone or in combination. The experiment involved 16 groups interacting for 24 h in microtubes. The groups (G1 to G15) containing any chemical or biological compound (NF34) or combinations thereof showed significant differences compared with the control group, except for G5, which contains 100% petroleum jelly without combinations. In that study, the authors reached the conclusion that the combined application of chemical compounds alongside biological control methods proved to be effective. However, they underscored the need for further research to better understand the synergistic interaction between chemical and biological agents, aiming at more efficient control of hookworms in the future.

In another study, Silva et al. (2022) [77] reported important results on the enzymatic “attack” of *D. flagrans* on eggs and second-stage larvae of *Toxocara canis*, a geohelminth that causes visceral larva migrans (VLM) in humans. The authors discovered that proteases and chitinases derived from the *D. flagrans* fungus were effective in destroying the cuticle of L2 stages. These findings suggest that the application of *D. flagrans* conidia/chlamydospores (AC001) may be useful in the future for controlling *T. canis* in contaminated environments such as plazas, parks, and community recreation areas.

In support of the work described above, Hao et al. (2024) [78] mentioned that the combined use of chemical products and helminthophagous fungi represents a sustainable strategy for livestock production, reducing costs, resistance, toxicity, and management, as well as reducing residues in animal products and the environment. The authors successfully recorded the activity of chemical (albendazole and ivermectin) and biological (*D. flagrans*) compounds on sheep gastrointestinal nematodes at different times. For humans, the fungi are saprophytes, and the carriers in formulations are inert or uncontaminated foods [32]. Hao et al. (2024) [78] observed that capsules containing the combination of fungus–ivermectin and fungus–albendazole were more effective in eradicating adults and L3 larvae than anthelmintics or biologicals alone. As a result, combining anthelmintics with helminthophagous fungi is a viable strategy that provides fundamental data for the advancement of biological control methods. In a recent work, Ferreira et al. (2024) [79] reported important results on a new possibility for the combined use of different species of helminthophagous fungi for the environmental biological control of eggs and *Taenia saginata*.

In the past, Zhang et al. (2018) [80] presented their perspective on a class of nanoparticles (NONPs) produced by the helminthophagous fungus *Arthrobotrys oligospora*, highlighting their unique properties for cancer immunochemotherapy. They suggested that fungus-based nanoparticles may represent a new approach to drug delivery for cancer therapy. According to Zhang et al. (2018) [80], NONPs can be used as cytotoxic agents, drug delivery systems, and immunostimulatory agents, all of which make them very useful in cancer immunochemotherapy. Recent studies by Brazilian groups, based on the work of Zhang et al. (2018) [80], have focused on the production of nanoparticles from the fungus *D. flagrans* (AC001) with anthelmintic activity and clear prospects for the production of bio-anthelmintics [71,78,81]. According to Barbosa et al. (2019) [71], most anthelmintics used to treat parasitic nematode infections act on target proteins or regulate the electrical activity of neurons and muscles, leading to paralysis, starvation, immune attack, and expulsion of the worm. However, current anthelmintics have limitations, such as a limited spectrum of activity across species and the threat of drug resistance, highlighting the need for new drugs for human and veterinary use.

Based on the premise that “the most common and practical way to use helminthophagous fungi under field conditions is through animal feed”, this important information could in the future be part of a safe strategic program for human parasite control. With the progress of current research, it is already possible to envision the practical viability of using helminthophagous fungi in two main areas: (1) a future bio-antihelminthic based on nanoparticles, and (2) a “synergistic model” combining chemical compounds (anthelmintics) and biological compounds (fungi) aimed at effective environmental control of preparasitic forms that could be used to “break” the helminth cycle in humans. Thus, research on these aspects could be the key to the practical application of helminthophagous fungi in the control of human geohelminthiases [77].

## 7. Conclusions

The practical feasibility of using helminthophagous fungi for parasite control of helminthic infections in humans is possible in the future, with the proposal of a commercial product that should be the “fruit” of the extensive scientific knowledge of the biology and safety of these organisms acquired over decades. The current literature supports the potential of these fungi as an environmentally friendly solution for managing helminthiasis and public welfare.

## Figures and Tables

**Table 1:** Use of helminthophagous fungi in the biological control of human-infecting nematodes and zoonotic intestinal parasites.

References	Fungi	Nemathelminths
[33]	*Arthrobotrys* spp.	*Ancylostoma duodenale*
[19,34]	*Pochonia chlamydosporia*	*Ascaris lumbricoides*
[70]	*Paecilomyces lilacinus*	*Toxocara canis*
[65]	*Pochonia chlamydosporia*	*Enterobius vermicularis*
[22]	*Monacrosporium thaumasium*	*Ancylostoma caninum*
[66]	*Duddingtonia flagrans, M. thaumasium, M. sinense* and *A. robusta*	*Ancylostoma ceylanicum*
[7]	*Duddingtonia flagrans*, *Monacrosporium thaumasium* and *Artrobotrys robusta*	*Strongyloides stercoralis*
[9]	*Pochonia chlamydosporia* and *Paecilomyces lilacinus*	*Toxocara canis*
[8]	*Pochonia chlamydosporia*	*Toxocara canis*
[67]	*Monacrosporium thaumasium*, *Monacrosporium sinense* and *Arthrobotrys robusta*, *Arthrobotrys cladodes* and *Arthrobotrys conoides*	*Angiostrongylus cantonensis*
[23]	complex *Trichoderma* and *Fusarium solani*	*Toxocara canis*
[68,71]	*Duddingtonia flagrans*	*Ancylostoma caninum*
[69]	*Mucor circinelloides* and *Duddingtonia flagrans*	*Toxocara canis*, *Toxascaris leonina*, and *Ancylostoma caninum*

**Table 2:** Control of platyhelminths in humans and zoonotic intestinal parasites with helminthophagous fungus.

References	Fungi	Platyhelminths
[16]	*Paecilomyces lilacinus*	*Taenia saginata*
[17]	*Duddingtonia flagrans*, *Monacrosporium sinense* and *Pochonia chlamydosporia*	*Schistosoma mansoni*
[18]	*Duddingtonia flagrans*, *Monacrosporium sinense* and *Pochonia chlamydosporia*	*Fasciola hepatica*
[20]	*Pochonia chlamydosporia, Duddingtonia flagrans* and *Monacrosporium thaumasium*	*Taenia saginata*
[21]	*Pochonia chlamydosporia* and *Paecilomyces lilacinus*	*Taenia saginata*
